# Proceedings of the 2013 CINP Summit: Innovative Partnerships to Accelerate CNS Drug Discovery for Improved Patient Care

**DOI:** 10.1093/ijnp/pyu100

**Published:** 2015-01-31

**Authors:** Anthony George Phillips, Peter Hongaard-Andersen, Richard A. Moscicki, Barbara Sahakian, Rémi Quirion, K. Ranga Rama Krishnan, Tim Race

**Affiliations:** Collegium Internationale Neuro-Psychopharmacologicum, Vancouver, British Columbia, Canada (Dr Phillips); Innovation Fund Denmark, Copenhagen, Denmark (Dr Hongaard-Andersen); US Food and Drug Administration, Silver Spring, Maryland, USA (Dr Moscicki); University of Cambridge, Cambridge, UK (Dr Sahakian); McGill University, Montreal, Quebec, Canada (Dr Quirion); Duke NUS, Singapore (Dr Krishnan); Deutsche Bank, London, UK (Mr Race)

**Keywords:** brain health, clinical neuropsychopharmacology, CNS drug pipeline, public-private partnerships, science policy

## Abstract

Central nervous system (CNS) diseases and, in particular, mental health disorders, are becoming recognized as the health challenge of the 21^st^ century. Currently, at least 10% of the global population is affected by a mental health disorder, a figure that is set to increase year on year. Meanwhile, the rate of development of new CNS drugs has not increased for many years, despite unprecedented levels of investment. In response to this state of affairs, the Collegium Internationale Neuro-Psychopharmacologicum (CINP) convened a summit to discuss ways to reverse this disturbing trend through new partnerships to accelerate CNS drug discovery. The objectives of the Summit were to explore the issues affecting the value chain (i.e. the chain of activities or stakeholders that a company engages in/with to deliver a product to market) in brain research, thereby gaining insights from key stakeholders and developing actions to address unmet needs; to identify achievable objectives to address the issues; to develop action plans to bring about measurable improvements across the value chain and accelerate CNS drug discovery; and finally, to communicate recommendations to governments, the research and development community, and other relevant stakeholders.

Summit outputs include the following action plans, aligned to the pressure points within the brain research-drug development value chain:

1. Code of conduct dealing with conflict of interest issues,
2. Prevention, early diagnosis, and treatment,
3. Linking science and regulation,
4. Patient involvement in trial design, definition of endpoints, etc.,
5. Novel trial design,
6. Reproduction and confirmation of data,
7. Update of intellectual property (IP) laws to facilitate repurposing and combination therapy (low priority),
8. Large-scale, global patient registries,
9. Editorials on nomenclature, biomarkers, and diagnostic tools, and
10. Public awareness, with brain disease advocates to attend G8 meetings and World Economic Forum (WEF) Annual meetings in Davos, Switzerland. In this context Professor Barbara Sahakian recently made a formal presentation at the World Economic Forum (see Barbara Sahakian Blog from April 11, 2014, at https://forumblog.org/people/barbara-sahakian/)

Full details of the discussions that formed the bases for these actions are presented in the main body of this document.

## Introduction

### Brain Disease: The Growing Challenge of the 21^st^ Century

Despite the enormous strides that have been taken in the understanding of central nervous system (CNS) diseases, healthcare systems worldwide face an unprecedented challenge in dealing with the unmet need associated with this disease area. CNS diseases, and in particular, mental health disorders, are the major health challenge of the 21^st^ century (Race et al., 2013). As argued cogently by Insel and Quirion (2005), there is an urgent need to establish a better balance and closer alignment between neurology and psychiatry. Currently, at least 10% of the global population is affected by a mental disorder, with up to 700 million people living with a mental disorder in 2010 (Patel and Saxena, 2014). It is estimated that a third of people will develop a brain disorder, with one in 20 people experiencing depression, and as many as one in a hundred developing schizophrenia (Race et al., 2013). According to the World Health Organization (WHO), the number of people living with dementia worldwide stands at approximately 36 million and is set to double by 2030 and more than triple by 2050 (see www.who.int/features/factfiles/dementia/en/#). Dementia exerts a huge burden on patients beyond the well-recognized symptoms of memory loss and cognitive decline: many sufferers experience emotional disturbances, including sadness, anger, and anxiety (see www.alzheimersresearchuk.org/dementia-symptoms/#acc1/).

The estimated global economic cost of mental disorders for 2010 was approximately US $2.5 trillion, with a projected cost for 2030 of US $6 trillion (Bloom et al., 2011). These figures are in line with WHO global and European studies that demonstrate that neuropsychiatric disorders represent a larger disease burden on society than either cardiovascular disease or cancer (Nutt and Attridge, 2014). The total cost of brain disorders—including mental disorders, neurodegenerative diseases, and malignancy—in Europe for 2010 has been estimated as €798 billion (Gustavsson et al., 2011). The breakdown of these costs is as follows: 37% direct healthcare costs; 23% direct non-medical costs; and 40% indirect social costs and productivity losses that are often overlooked.

### Barriers to Developing New CNS Drugs

The majority of people are unaware of the prevalence of brain disease, in particular mental illness, and its burden on society, not least because of the stigma surrounding those brain diseases that give rise to mental ill health. Drawing this distinction between the stigma associated with psychiatric disorders rather than brain diseases in general underscores the injustice of assigning stigma to these specific brain disorders. There is reluctance for society to engage with the issue of mental health disorders and also a paucity of mental health advocates; by the very nature of their condition, mental health patients are often unable to help raise awareness. The net result of this resounding silence on mental health is that the area has been increasingly ignored from an investment perspective by a significant section of the pharmaceutical industry and financial market investors.

To respond to this challenge, companies and academic researchers need to be able to exploit advances in science and medicine to develop innovative medicines. Improved therapies for serious brain disorders would positively impact quality of life (QoL) and ability to function for those affected, leading to improved labor productivity and a reduction in healthcare costs and overall burden on society. However, the CNS drug pipeline is beset by a series of key challenges that must be urgently addressed.

Although ever more knowledge is being acquired regarding the aetiology and mechanisms of CNS disorders and the potential therapeutics for these disorders, this knowledge has yet to be put into practice in many cases (Manji and De Souza, 2009). Perceived barriers to stimulating the CNS pipeline include the unparalleled complexity of the CNS itself, lack of a defined disease pathology in most cases, little or no direct access to tissue for research, plus the fact that many CNS diseases manifest themselves as abnormal behaviors that are difficult to characterize and assess, with ratings scales and questionnaires substituting for clearly defined endpoints. There is also a lack of understanding of the molecular basis for many CNS disorders and insufficient interdisciplinary research collaborations that would channel the expertise of different but related specialists towards common research goals.

Other reasons for the failing CNS pipeline include extended drug development timelines, increased drug development costs, and higher risk of clinical failure. It has been estimated that as little as 8% of potential CNS drugs actually make it to clinical use, compared with 15% for candidate drugs in other areas of medicine (Riordan and Cutler, 2011). It also takes substantially longer for CNS drugs to achieve regulatory approval; an estimated 1.9 years for CNS drugs, compared with 1.2 years for non-CNS drugs (Riordan and Cutler, 2011). Furthermore, Phase II and III development takes an average of 8.1 years for CNS drugs—2 years longer than for drugs in other areas of medicine (Riordan and Cutler, 2011).

The discouraging scenario outlined above may go some way to explaining why pharma has been changing its strategies for drug development within the CNS arena in recent years. Clearly we are in a period of significant change in which at least three global pharma companies have announced closure of their neuroscience divisions worldwide in 2011, with four others significantly downsizing CNS operations (Skripka-Serry, 2013). Offsetting these events, many other companies have committed significantly more resources to this area (e.g. Lilly, Pfizer, Lundbeck, Roche, Astellas).

### Opportunities to Benefit from New Drug Development

Given the global prevalence of brain disease—350 million people living with depression, 70 million with schizophrenia, and 36 million with dementia—and the prospect of an aging population making ever greater demands on healthcare provision, there is a clear opportunity for the pharmaceutical companies to enter the field successfully. Meanwhile, despite the assumption that therapeutic targets in brain disease have been fully investigated, this is not the case and there is still much to do in the area of researching the basic biology of the brain. To this list one should add that despite very compelling preclinical data, many large academic efforts to improve drug treatments have also failed to yield significant outcomes. It is accepted that many current drugs have serious flaws, providing significant opportunity to develop compounds based on new targets that address the issues of low efficacy and poor tolerability.

### CINP Activities to Overcome Barriers Within the CNS Drugs Pipeline

In light of the dwindling CNS pipeline and huge unmet need for improved treatment of brain diseases—and against the backdrop of weak global economic performance—the CINP held its inaugural Think Tank in Munich, Germany, in 2012. Bringing together delegates from disparate clinical, research, and industrial backgrounds, this small, open meeting was intended to discuss the barriers to developing new drugs for brain disorders and identify useful approaches to overcome these barriers (Dean et al., 2014).

The 2012 inaugural CINP Think Tank recognized that there were drivers and barriers influencing the development of new drugs for psychiatric disorders and concluded that:

- Although understanding the core pathophysiology of brain disorders may not necessarily lead to the development of new drugs, it is a fundamental step in the right direction. Therefore, it is essential to progress research on the neurobiological bases of brain disorders.
- Understanding the cause of a disorder may not deliver new drug targets since there may be no pharmacologically accessible target or the primary event leading to the onset of illness may be decoupled chronologically.
- Developing biomarkers for brain disorders is critical to identifying subjects at risk of a disorder, improving diagnostic consensus, and providing early indications of pharmaco-responsiveness. However, as no validated biomarkers are yet agreed upon, other surrogate markers of efficacy are needed
- There is a need to accelerate the development of behavioral models that allow findings on drug indications and efficacy to be accurately translated from animals to humans.
- Understanding the primary mechanism of action of any psychotropic drug may not pinpoint the cause of psychiatric disorders.
- To successfully understand the full potential of new drugs a greater emphasis is required within experimental medicine to encourage creative clinical investigations and improved communication between preclinical neuropsychopharmacologists, clinicians committed to neuropsychopharmacological research, the drug industry, and regulators.

It was agreed that the CINP must continue its role as a conduit between industry and academia as a central component in the identification of new drug targets, development of new drugs, and delivery of these drugs to the clinic.

Other international organizations, in collaboration with the CINP, have begun to address

ways of promoting a greater awareness of mental health issues to the general public. Currently, there are two initiatives in place focused on engagement with and advancement of mental health within the working populations of Europe and beyond. Chaired by Bill Wilkerson (Mental Health International, Toronto, Canada), *Target Depression in the Workplace* is a pan-European employer initiative intended to create a unified front of employers to reduce the effects of depression on the wellbeing and productive capacity of working men and women. Currently, some 16 employers, representing nearly one million people in Europe and around the world, have become members of the Human Resources Leadership Forum to Target Depression in the Workplace. A second major initiative involves the USA/Canada Forum on Mental Health and Productivity, the fifth meeting of which took place in Toronto, Canada, in November 2013 (Wilkerson, 2014). The outcome was a consensus that business must become a strategic partner of brain science in pursuit of real answers to those conditions concentrated so heavily among working populations.

In addition, the European Commission and the Irish presidency of the European Union (EU) organized the conference “Healthy Brain: Healthy Europe—A new horizon for brain research and healthcare in Europe,” which took place May 27–28, 2013, within the framework of the European Month of the Brain. Key conference objectives were to encourage public authorities to develop and coordinate national brain research and healthcare strategies; to help lift taboos associated with brain health issues, including mental disorders; to facilitate the absorption and integration of research results into policy and good practice; and to advance the practical application of a new paradigm of brain research and healthcare, taking into account patient needs.

### CINP 2013 Summit Meeting

Following on from the success of the 2012 CINP Think Tank and the European Month of the Brain 2013, which saw the organization of over 100 events in EU Member States and Associated Countries, the CINP held its “2013 CINP Summit: Innovative partnerships to accelerate CNS drug discovery for improved patient care” in Munich, Germany, the outcomes of which are the subject of this document. The Summit had the following overarching objectives:

- Identify achievable goals to address issues relating to CNS drug discovery across five key topics:
  1. Connecting science and regulation,
  2. Benefit–risk, effectiveness research, and implementation in clinical practice,
  3. Knowledge transfer and protection of innovation,
  4. The need for a modern, 21st century perspective on new tools for assessing treatment effects, and
  5. Incentivizing investment in brain research.

- Develop action plans to bring about measurable improvements across the value chain and accelerate CNS drug discovery (see Table 1).
- Communicate recommendations to relevant stakeholders globally.

**Table 1.** Key Action Items Proposed at the 2013 CINP Summit Meeting in Munich

	Action Categories	Goal	Action
1.	Code of conduct dealing with conflict of interest issues	To develop a global code of conduct, making it possible for stakeholders in healthcare systems to work together and facilitate healthcare innovation.	Initiation of a working party with representatives from patient and caregiver organisations, from industry, from scientific and medical organisations and from payors – and jointly led by the PMDA, FDA and EMA – to develop a code of conduct for any type of collaboration between the parties. The Code should be globally accepted and should build on already established codes in the healthcare area.
2.	Prevention, early diagnosis and treatment	• To reduce the number of people developing depression • To provide support for people with schizophrenia living with their families and in their normal social context • To improve QoL of patients suffering from neurodegenerative disorders, ease the burden on caregivers and delay nursing home admittance by diagnosing and treating early	• Depression: Undertake a major initiative to develop tangible recommendations to be implemented in human resources (HR) policies within the workplace. Recommendations should be developed in collaboration with relevant stakeholders, including employers and employee organisations and should be modelled on the Europe-wide initiative ‘Target depression in the workplace’ or Mental Health Canada (Wilkerson, 2014) • Schizophrenia: Undertake an initiative around early diagnosis and treatment to allocate social support to maintain patients with schizophrenia within the context of their family and social network, ideally allowing them to complete their education. The initiative could be based on the Danish Opus project (Andreasen, Opus Project, see www.ispn-psych.org/docs/Opus_Project.pdf). • Neurological conditions: Initiate a public awareness campaign to make patient and caregivers/family members aware of the symptoms and the benefits of early diagnosis and treatment, including improved QoL and reduced burden on patients and caregivers alike.
3.	Linking science and regulation	To double the number of CNS drugs in the pharma development pipeline by 2018.	Create an international forum on brain diseases involving the patient communities, FDA, EMA, PMDA and representatives from the payor community and led by the CINP to facilitate: • Co-ordination of payor and regulatory requirements • Accelerated translation of scientific breakthroughs into new endpoints and assessment tools acceptable to regulatory bodies • Assessment of the potential impact of scientific paradigm shifts and latest scientific developments on clinical trial development
4.	Patient involvement in trial design, definition of endpoints etc	• To increase the relevance and impact of R&D • To improve translation and back-translation methodology	• Engage with leading national and international patient organisations within the scope of CINP activities to create a dedicated international platform • Develop guidance for researchers on involvement of patients, regulators, payors etc in planning, execution and exploitation of R&D • Engage with projects aiming at redefining benefit–risk assessment methodologies as well as collection of real-world data (e.g., IMI or Horizon 2020) (see www.who.int/ medicines/areas/priority_medicines/en/)
5.	Novel trial design	To decrease number of patients and duration of clinical trials while increasing robustness of evidence generated.	• Mapping of adaptive design initiatives to identify gaps that can be addressed through collaborative research in the space of brain diseases and new models that would satisfy regulatory and payor requirements • Engage regulators and clinicians in dialogue to explore ways to speed up acceptance of adaptive trials • Engage with electronic health records/IT community to explore ways to strengthen and harmonise the data collection and processing infrastructure (including quality and standards of data) • Promote adaptive trial design in the clinicians’ community
6.	Reproduction and confirmation of data	To increase the overall quality of scientific and clinical data.	Engage with publishers, public and private funders of research to discuss the potential solutions to the lack of reproducibility.
7.	Update of intellectual property (IP) laws to facilitate repurposing and combination therapy	To facilitate research on novel and old compounds to fully exploit their therapeutic potential.	To map current incentives vs unmet needs to identify opportunities/gaps within the current system. This work should be carried out by a PPP combining the efforts of academics, industry, patients, regulators and payors. As a global organisation, the CINP is well positioned to lead this activity.
8.	Large-scale, global patient registries	To set up high quality patient registries in priority areas.	Define an action plan based on mapping and analysis of current registries for filling the gaps. The action plan should be run as a collaborative project between patient organisations, industry and the public health systems, potentially as a PPP.
9.	Editorials on nomenclature, biomarkers and diagnostic tools	To have globally accepted nomenclature for brain diseases – ideally linked to defined biologies and criteria for biomarkers for brain diseases.	• An editorial on common nomenclature was published in 2014. Prof. Zohar (ECNP, Israel) was responsible for this action as an important part of the effort to accelerate CNS drug discovery (Zohar et al., 2014) • An editorial for publication in 2014/2015 outlining the usefulness of biomarkers in psychiatry and the development of other types of diagnostic tools, including cognitive and electrophysiological markers. This action will be led by Prof. Kapur (King’s College London, UK) and Dr Zoran Simic (Medicines and Healthcare Products Regulatory Agency (MHRA), UK)
10.	Public awareness – brain disease advocates to attend G8 meetings and World Economic forum (WEF) annual meetings in Davos, Switzerland	• To promote the recognition and prioritisation of brain disorders • To stimulate investment in brain disorders across healthcare systems and in basic research, commensurate with the societal burden of these conditions	• Initiation of global awareness campaigns led by the CINP, EBC and WHO • Attendance of brain disorder advocates at G8 and WEF meetings from 2017

The CINP Summit attendees included representatives from regulatory bodies, payors, academia, and industry; together they addressed the above five key topics.

### Topic 1: Connecting Science and Regulation

Defining disease has always been challenging in brain disorders, with very few biomarkers being successfully applied. If a molecular definition of a range of illnesses,

such as depression, could be applied and biomarkers developed, treatment would advance more rapidly. For example, it might be that a drug used to treat depression is actually only targeting one of 10 causes of the disorder. Improved diagnostics would help to better understand how the drug was working and explain different components of the disease. This could, in turn, lead to development of drugs that could be used to treat a component of depression, for example, cognitive dysfunction.

With the current dearth of new drugs in the CNS pipeline, there is a clear need to seek alternative avenues for much-needed drugs to be brought to market. Creating a more collaborative and open approach to drug development may allow researchers and others to seek input from regulatory authorities and payors, thus enabling the potential of new therapies to be investigated. Currently, there are many misconceptions around the role and attitudes of regulatory authorities. The field of CNS research will benefit considerably from a more collaborative style of communication between stakeholders in the value chain. Dialogue between regulatory authorities, payors, and researchers will help to enhance the way clinical trials are carried out.

By focusing on understanding disease pathways through basic research, it should be possible to elucidate the molecular mechanisms that lead to disease. A clearer understanding of underlying disease mechanisms will facilitate an improved classification of diseases and more sophisticated potential treatments.

One of the key challenges to initiating basic research is attracting investment. For this to happen, it is critical that investors, researchers, regulators, and payors should achieve a greater level of collaboration, aligned with a better understanding of the needs of their collaborators.

Another key challenge is to ensure that scientific advances are translated into refinements in regulatory processes. For instance, new clinical endpoints and measurement scales must be reviewed and evaluated for acceptance by regulatory bodies for inclusion in pivotal studies. To achieve this, regulators will need to be convinced of the robustness and validity of any new endpoints or measurements, and payors will require evidence of the positive health and economic implications of adopting these new methodologies. Regulatory reviewers and research scientists all need to stay abreast of advances in the health sciences to ensure they can reach consensus on improving clinical trial parameters. Bodies such as the International Conference on Harmonisation have a key role to play in bringing together the regulatory authorities and pharmaceutical industries of Europe, Japan, and North America to discuss scientific and technical aspects of drug registration.

Public-private partnerships (PPPs) for the funding of drug development can be encouraged by creative initiatives, such as the Food and Drug Administration’s (FDA) Critical Path Initiative (CPI), launched in 2004. The CPI is the FDA’s national strategy for modernizing the sciences through which FDA-regulated products are developed, evaluated, manufactured, and used. Currently, the FDA participates in many PPPs, with eight that have been developed in collaboration with the CPI. Many of the projects involve partnerships among FDA centers and between the FDA and other organizations, including other federal agencies, academia, patient advocacy groups, and the drug industry.

To maintain the quality and relevance of a PPP network, organizations such as the CINP, American College of Neuropsychopharmacology, and European College of Neuropsychopharmacology (ECNP) should collaborate with governments and the drug industry to review and coordinate PPP activity.

Other FDA mechanisms that enhance external input into FDA decision-making include special government employees from academia to provide independent and objective advice to the FDA, advisory committees, workshops, working groups, and the Voluntary Exploratory Data Submission mechanism.

These FDA mechanisms are conduits that allow researchers to access the FDA, which traditionally is perceived as disconnected from academia. Part of this misalignment is based on the fact that academic researchers do not know or understand the requirements and processes of the FDA, while the FDA has historically been less engaged in academic research data. Data from academic research has often proven difficult to reproduce and is not subject to the same scrutiny that industrial work often is, leading to some regulatory concerns regarding its validity.

It should be acknowledged that while the FDA has set up some innovative and forward-thinking organizations, they do not necessarily lend themselves to the area of neuropsychopharmacology. By their very nature, they are silos of endeavor and do not give a comprehensive view of the CNS field. It was proposed that a broader forum should be set up to consider neuropsychopharmacology, with a particular focus on developing new primary endpoints and measurement scales.

In Japan, the relatively recently formed Pharmaceuticals and Medical Devices Agency (PMDA) currently gives similar consideration to oncology as it does to CNS drug development. However, while cancer drug development is based on clearly-defined endpoints, CNS drug development relies on assessment scales and observation. Furthermore, CNS diseases are far more complex than those in oncology and less well understood. It will take time and patience to raise the understanding of CNS disease to the same level as other fields of medicine.

The PMDA forms science boards, which include external members to better discuss innovative areas and challenge reviewers’ experiences. Consultation with academia is at the heart of the regulatory process. Medical devices and drugs are both considered by the same processes, with face-to-face consultation services, such as clinical-trial consultation, and advice on application materials before the clinical trial stage.

The current situation is less evolved in Europe, where the European Medicines Agency (EMA) has a more conservative approach to engaging with pharmaceutical companies, which could lead to poor communication and uncertainty and have a negative effect on drug development. The EMA had withdrawn from meetings because of concerns about conflicts of interest. However, more recently it has relaxed its position and is now collaborating with the ECNP. The absence of regulators from advisory committees and other key industry and academic meetings may have a detrimental effect on the dialogue between scientists and regulators and, in turn, hinder research efforts. In this context, perceived conflicts of interest represent a considerable barrier to specific drug development, but one that can be overcome by risk-sharing initiatives.

In Europe, the largest PPP is the Innovative Medicines Initiative (IMI), which supports more efficient discovery and development of more effective and safer drugs. The IMI is a joint undertaking between the European Commission and the European Federation of Pharmaceutical Industries and Associations. With a €2 billion budget, IMI

supports collaborative research projects in the areas of safety and efficacy, knowledge management, and education and training, and builds networks of industrial and academic experts in Europe to boost innovation in healthcare. Apart from partners from industry and academia, research consortia include representatives from patient organizations, hospitals, and regulatory agencies. Many of the IMI projects are relevant for CNS drug development, including mental disorders, and will be important for revisiting the regulatory environment in light of the particular challenges in this area.

Paying close attention to priorities defined by regulatory authorities and unmet needs expressed by patients and caregivers provides a solid foundation for a renewed PPP, the IMI2, under Horizon 2020, the European Framework Programme for Research and Innovation 2014–20. The Strategic Research Agenda of IMI2, based on the WHO’s 2013 “Priority Medicines for Europe and the World” report (see www.who.int/medicines/areas/priority_medicines/en/), reflects the major challenges facing the European healthcare systems, the pharmaceutical industry, and the regulatory framework and clearly identifies psychiatric and neurodegenerative diseases amongst the priorities to be addressed. To ensure that research remains cutting-edge and public funds are used in the most efficient way, collaboration with other large international PPPs will be key.

It was strongly suggested that mechanisms should be put in place for European payors and regulators to ensure they are kept updated on science breakthroughs and new trends. Furthermore, funders and journals should have higher publishing standards to maintain the quality of the scientific research that is used to develop new biomarkers and methodologies.

Currently, North American academia is increasingly becoming a source of new ideas for CNS drug development and has a role in the transfer of these ideas into products. Indeed, one is also seeing increasing examples of new partnerships between academia and both smaller biotechnology companies as well as larger pharmaceutical enterprises. However, for this model to succeed, the academic community must be aware of its role in the process, and needs to present data at the appropriate points in the process and in a way that is acceptable to the FDA. While this concept found early adoption in the USA, the above paradigm is becoming a global phenomenon in reaction to the widescale changes currently underway throughout the world as large pharmaceutical companies adapt to the significant challenges of developing new and cost-effective therapies for a wide range of brain-related disorders.

Regulatory authorities are reluctant to embrace new methodologies without substantial data to qualify their use. A case in point is the relatively new paradigm of depression as a change in the circuitry of the brain rather than a chemical imbalance, and hence something that might respond to therapies other than chemicals. Although this model has found favor among researchers, regulatory bodies would require a significant amount of proof before they would accept this view. It is exactly this burden of proof required in order to gain acceptance of a new paradigm that dissuades large pharmaceutical companies from entering the arena of CNS research. In order to counter this lack of investment, a combination of improved diagnostics, investment from PPPs, and closer collaboration between industry, research, and regulators must be encouraged.

The current diagnostics classification, the Diagnostic and Statistical Manual of Mental Disorders-5 (DSM-5; American Psychiatric Association, 2012) has been described as at once indispensable and unhelpful. The Research Domain Criteria project has been launched by the National Institute of Mental Health to develop, for research purposes, new ways of classifying psychopathology based on dimensions of observable behavior and neurobiological measures. The project aims to be more practical and relevant than the DSM-5 and translate rapid progress in basic neurobiological and behavioral research into an improved understanding of psychopathology, leading to the development of new treatments for brain disorders. Adoption of the Research Domain Criteria process, including a redefinition of brain disorders, by regulatory bodies such as the FDA and by payors could pave the way for a more flexible approach to drug development.

### Topic 2: Benefit-Risk, Effectiveness Research, and Implementation in Clinical Practice

Today’s clinical trials are typically carried out in a setting that differs significantly from real-life medical practice. Trial participants are recruited on the basis of narrow criteria designed to provide the ideal population for the treatment being studied. Generally, patient populations are filtered with regard to age, lack of comorbidities, and potential to respond to treatment. Furthermore, co-medication, adherence to the study drug, and reporting of adverse events are all rigorously overseen in a manner that does not reflect real-world clinical practice. Nevertheless, traditional randomized, placebo-controlled trials are generally still demanded by regulatory authorities in order to assess suitability for licence approval.

The challenge for those working in the field of CNS drug development is to present new and credible clinical trial designs to regulators and payors in a way that ensures they are accepted as valid methodologies on which to base approval decisions. Innovative trial designs with relevant and reliable endpoints are essential for the evolution of a system of drug development that brings new treatments to market faster and at lower cost than at present, without adversely impacting acceptable safety standards. An additional benefit of any such new trial design would be to make the drug development process more predictable.

Discussion must be focused on how to identify methods that would ensure real-world effectiveness are properly evaluated and become a core component of a more adaptable system for approval, pricing, and reimbursement assessment of new therapies in the CNS field. Specifically, consideration should be given to mechanisms for increasing the importance of patient-reported data in effectiveness evaluations and incorporating assessment of patients’ benefit-risk perception into trial designs.

A key challenge for drug development is to explore new concepts for ensuring that new treatments are properly remunerated. In situations where the percentage of patients potentially able to benefit from a drug is high (e.g. 60–70% of patients able to respond to a medication), manufacturers should consider the implications of this for payors in terms of cost-benefit comparisons and negotiate drug costs appropriately. However, the situation is complicated by the increasing need to adopt more patient-centric approaches and consider specific drug treatments within the context of a holistic package of interventions.

In Germany, cost-benefit is particularly important in determining the availability of a new therapy. This model requires the involvement of payors who decide whether a technology is worth investing in as part of the health technology assessment (HTA). In Europe generally, there is an HTA network that informs pricing and reimbursement decisions. The HTA is a way of assessing how technologies are used in healthcare and disease prevention. It covers medical, social, economic, and ethical issues in a transparent, robust, and unbiased manner.

When considering development of drugs from Phase I to Phase IV trials, there are certainly opportunities for improvement. Animal studies should be carried out using sound methodology and an understanding of whether the desired effect is to treat a whole disease or instead focus on essential features of that disease: i.e. attempting to treat schizophrenia or improving memory and reducing apathy in patients with schizophrenia; attempting to treat attention deficit hyperactivity disorder (ADHD) or reducing impulsivity and improving attention in affected patients. Defining a measurable effect means that a comprehensive animal model of disease is not necessary and should also ensure that the early animal research can translate into human studies.

Careful consideration of benefit-risk calculations and inclusion of patient perspectives in trial design may be a way to reduce the financial risk of trial development. The key challenge is identifying current sources of data and harnessing new sources of relevant information that could contribute to a better understanding of a drug’s performance.

One of the most extensive changes explored is the involvement of patients and patient advocate groups at the study design level. Patient involvement could be expected to drive recruitment, improve access to existing directories, and define the outcomes that are captured during the trial itself. Such a collaborative approach would have the added benefits of encouraging further patient engagement and ensuring that the patient voice is heard.

A key benefit of involving patients and advocates at the trial design level is that they can influence the choice of the outcomes measured and establish real-world targets against which drugs can be assessed. Such an approach would prove invaluable not only to pharmaceutical companies and payors, who could better assess the true value of a drug, but also to other stakeholders, including regulators, healthcare policymakers, healthcare professionals, and patients.

An historic example of patient advocacy contributing massively to the advancement of disease understanding and drug development is the field of HIV and AIDS. The disease has gone from being an incurable, deadly infection to a manageable, chronic condition in the space of 30 years. That the picture changed so rapidly is due in no small part to the contribution of a vocal and committed patient population, who embraced everything from fundraising to activism to demand better treatment. However, it should be remembered that many people living with brain disorders may find it more challenging to organize an effective response to the poor state of drug development in the CNS arena than was seen with HIV/AIDS.

In the past, translational studies involving primates have been essential to development of CNS drugs. However, the increasing difficulties in obtaining licenses to carry out CNS studies on primates, which exhibit behaviors and conditions similar to human brain disorders, is impeding the development of novel CNS drugs. Rodent models of brain disease are inevitably limited, as the animals do not have the same levels of mental function as humans. Nevertheless, there are similar brain pathways and certain behaviors that are indicative of pathway effects that correspond to human brain disorders. Better design of animal studies will maximize the utility of available animal models and reduce the proportion of drugs failing in Phase II or III trials.

The importance of including assessments of functionality in future clinical trial design was also discussed. The fact that the WHO had been instrumental in adding a requirement for functional assessment into the recently published fifth edition of the DSM-5 (American Psychiatric Association, 2012) is an indication of how seriously the issue of patient functionality is now being taken. The inclusion of functional outcomes in clinical trials will also mean that governments will be more inclined to fund a novel treatment where functional benefits to patients have been demonstrated. There is ever-increasing awareness that returning an individual to a level where they can once again cope with everyday activities holds the very real promise of reducing the costs for healthcare and social support systems. Further benefits would accrue when these individuals resume or take up employment, thereby contributing to the national economy. The same is true when a child with a brain disorder achieves a functional level that allows an adult family member or other caregiver to return to full-time employment.

The development of methodologies that reflect the patient voice must involve assessments that are robust and practically attainable. These methodologies must be patient-centric and patient-focused, with a clearly identifiable benefit-risk profile. A key challenge with this patient-centric approach is the harnessing of real-life data to facilitate the timely approval of a drug with well-defined efficacy and safety data. Improved real-world data collection could be driven by use of technology, such as smart phones, other smart devices, and the internet (also known as e-health and m-health).

Incorporating patient-reported real-world data into clinical trials will allow access to new types of evidence, and provide answers on real-life benefit-risk considerations. However, it should be recognized that even well-thought-out methodologies using self-reporting will inevitably provide outcome data that are subjective. Much care will be required to separate “signal” from “noise” and obtain robust and applicable outcomes.

Although the ideal scenario would be to design and populate a “bespoke” database that incorporates all the desired elements, including new methodologies and real-world clinical data based on patient-centric endpoints, the barriers to the creation and utilization of such a database are considerable. Legal, ethical, and regulatory requirements vary widely between countries and the cost of setting up a large database would be prohibitive. Alternative approaches include designing smaller-scale, interoperable databases that would allow data to be compiled and compared between different countries or the interrogation of existing databases for relevant data.

### Topic 3: Knowledge Transfer and Protection of Innovation

Intellectual property (IP) rights are applied to virtually every area of scientific endeavor. In universities, scientific innovations which have already received public funding are privatized and resold to the public via patents acquired by commercial interests. Clearly, there is a need to protect scientific achievement and encourage research. However, the fruits of this scientific endeavor need to be delivered more efficiently and cheaply to those in need, and this presents a challenge. We need to consider how to balance the need to share knowledge while also protecting IP.

The development of a framework within which industrial and academic scientific advances could be disseminated to the wider public without compromising commercialization and return on the developers’ investment was discussed. There was a focus on mechanisms for organizations, such as the CINP, to work closely with academia to improve IP processes so that others could benefit from scientific discoveries before IP becomes critical. Opinions were also sought on what might be perceived as appropriate incentives to protect new developments by IP processes in academic research, and whether there were opportunities to form PPPs to drive knowledge sharing.

Limited information sharing was not perceived to be the key issue, but instead concern focused on the disturbing trend that too much of the information published cannot be replicated (Landis et al., 2012; Prinz et al., 2011). One way to address the shortcomings of knowledge sharing between industry and research institutes is to consider the widely differing organizational cultures prevalent in these two sectors. Universities and academic institutes are primarily concerned with discovering and disseminating new knowledge while, conversely, pharmaceutical companies are focused on harnessing knowledge to cure or treat diseases for financial gain. However, since passage of the United States Patent and Trademark Law Amendments Act of 1980 (Public Law 96–517), universities have become increasingly aware of the advantages of protecting and managing their IP.

Researchers and administrators working in technology transfer departments in academia tend to overvalue early-stage IP and have the perception that by patenting a new technology in the early stages of its development they are increasing the chances of that technology being successfully adopted by industry. Meanwhile, those in industry are less concerned with patenting early-stage IP and more interested in protecting the commercial rights of a future, fully developed product. It was generally agreed that academia should greatly reduce the emphasis on IP rights because of the increased burden of bureaucracy, but others countered that continuing dialogue between the two sides was a more healthy approach. Where there is abundant collaboration between industry and academia there is the opportunity for either side to positively influence the other.

Rather than academia viewing industry as a source of funding and industry seeing academia as a good investment, adoption of a more collaborative, flexible, and open-ended approach might lead to improved drug development. All too often cultural barriers remain in place that may result in academic researchers who collaborate with industry being ostracized from academia in some countries.

Alternatives were discussed, such as ways in which companies could take and develop ideas from academia, then give the IP back to the original researcher/institute if an idea is developed successfully. A new approach is required to allow the academic researcher or institution to retain certain IP rights while increasing the incentive for industry to develop the technology. Such an approach was deemed to be feasible. However, there is currently too high a noise-to-signal ratio, and filter mechanisms would need to be developed to ensure that industry can identify potentially commercial technologies more reliably.

It is important to find the levers that will encourage academia to share their knowledge. If there is no incentive for an academic to contribute to a pharmaceutical company’s drug development then they are unlikely to do so, so there is a need to incentivize around ideas and to reward reproducibility. Some may feel that the insistence on reproducibility can itself become a barrier. While it is natural that a researcher who has developed a technology or a new molecule will want to move on and address the next intellectual challenge, it is imperative that the highest priority be placed on the need in industry and regulation to have the assurance that novel findings can be replicated. Currently, there is a one-way street where the onus on replication often falls to those colleagues in industry who wish to utilize new techniques and discoveries, and this needs to change. Although there is a focus on publishing research in well-respected journals to attract future grants, there is very little incentive for researchers to repeat experiments to demonstrate reproducibility. Additional funding to facilitate repetition of experiments might address this issue. A grant program could be developed to reward replication of data, which would then make the technology being developed more attractive to industry and valuable to society.

At this stage, collaboration between industry and researchers could help to push ideas forward and reassure academics that their IP rights will be respected once the technology is at a more advanced stage of development. This would remove the barrier of early IP rights and bring those rights into play at a more relevant stage, thus providing benefits to both sides of the partnership.

It was concluded that the best way to encourage open and mutually beneficial collaborations between academia and industry was to have continuous, frequent, well-structured meetings for the exchange of ideas before IP rights had been asserted. There are now many examples of companies that have benefitted from the open exchange of ideas with academia. Indeed, Cambridge, MA, has become a hub that attracts many commercial enterprises, ranging in size from small start-ups to large integrated pharmaceutical companies, due to the possibilities of open interactions with local academic institutions. Novartis is an excellent example of a company moving in that direction; however, other companies have already set this in motion (Astra Zeneca, Pfizer, Biogen) by establishing fruitful interactions between their Cambridge scientists and the local academic community. These pharma-academia interactions provide excellent examples of new approaches and partnerships that are helping to solve the issues raised in this manuscript.

There is a clear need for academia-industry collaboration to be increased and risk reduced. This need is already being addressed in other areas of research: for instance, the aerospace industry has a meeting for all the involved companies every two years. The Canadian Consortium for Research and Innovation in Aerospace in Québec promotes collaboration between industry specialists and researchers to identify and implement precompetitive projects that meet industry requirements. However, some obvious barriers to such a meeting in CNS research need to be recognized. Firstly, researchers are likely to be reluctant to discuss their own technological discoveries that are not yet protected by IP rights. Secondly, unproven technologies are unlikely to be attractive to industry, although the example of the aerospace industry shows that such barriers are surmountable. It was proposed that adopting a similar approach in the area of CNS research by encouraging a consortium or network of companies working together to reduce the risk might lead to better outcomes, including streamlining of IP rights acquisition and improved data access. However, with the noticeable recent withdrawal of some large pharmaceutical companies from the CNS field, there is a concern that such a consortium may not be achievable. Geography is a further complication, with international cooperation being a key element. Unfortunately, IP is dealt with differently in different countries, which may complicate large network collaborations.

Another method to spread risk is to foster PPPs, with not just one PPP working in isolation, but larger groups and networks of PPPs. Academic and industrial stakeholders could meet in cooperative research centers, each with its own board. Each group would contribute an investment stake and then the cooperative as a whole would approach the government. If the government approved a proposal, they would contribute an equal sum of money to the total cooperative investment stake. The obvious benefit to industry partners is that where an investment is successful, they will each get a considerably larger return on their original stake than would ordinarily be expected. The wider but less immediately obvious benefit is that where these cooperative research centers succeed, they tend to accumulate strong clinical ideas. Once an institution develops a reputation for being a center of excellence, they become more attractive to industry investors, and hence a “virtuous circle” is created.

### Topic 4: The Need for a Modern 21st Century Perspective on New Tools for Assessing Treatment Effects

The formal assessment of treatment effect and clinical status of patients with brain diseases in clinical trials is typically based on clinical scales and tools developed in the 1970s and ’80s. This situation is dictated by the requirements of various regulatory bodies for standardized and accepted tools for measurement of safety and efficacy of the treatment under investigation. Inevitably, these clinical scales do not incorporate the latest disease insights and understanding. Accordingly, we now face a paradox that has serious implications for innovation and working towards a more efficient and relevant approach to CNS drug development: some of the conventional clinical tools may no longer be clinically valid, but they remain at the core of regulatory processes.

In parallel to new insights driving the need for improved assessment scales, the enhanced understanding of the complexity of brain disorders has led to an acceptance that many named conditions or diseases are actually syndromes. Hence, it is perhaps not surprising that efforts to identify specific biomarkers have not been as productive as hoped, and their adoption as accepted clinical measurement tools has been limited.

Discussions were undertaken to identify methods to encourage research and development into new clinical assessment tools, such as clinical rating scales and biomarkers, to better serve the needs of CNS drug developers, regulators, and patients, and facilitate the acceptance of these tools.

Discussions began with agreement that there was much confusion around the common nomenclature, especially in the field of psychiatry, with different terms being used interchangeably and sometimes inappropriately. Continuing to work in a discipline where terminology is muddled and misleading can only lead to further confusion and wasted effort. A standardization of the arbitrary use of language that currently exists would help to establish consensus and unify research and clinical activity.

Much of psychiatry uses assessment scales that originated in the 1970s. These scales were based on small patient numbers and may not reflect patient function at all. Barriers to developing newer assessment scales include lack of recognition by regulatory bodies and consequent lack of enthusiasm from industry to invest financially in developing and validating such scales. Collection of long-term data that are clinically meaningful and relevant to patient function might help in the development and testing of more suitable assessment scales. The great advantage of large-scale data collection over a randomized, clinical trial is that real-world data would be generated as opposed to the data from an artificial randomized, controlled trial population. Gathering such large-scale, real-world data over the long term could identify trends and associations that may improve the understanding of brain disease.

A similar scenario was noted with regard to the utility of biomarkers in the field of psychiatric disease. Biomarkers are described according to characteristics that are objectively measured and evaluated as an indicator of normal biological processes, pathogenic processes, or pharmacological responses to a therapeutic intervention. They can be broadly categorized into three different types:

- Type 0 biomarker: A marker of the natural history of a disease that correlates longitudinally with known clinical indices.
- Type 1 biomarker: A marker that captures the effects of a therapeutic intervention in accordance with its mechanism of action.
- Type 2 biomarker (surrogate endpoint): A marker that is intended to substitute for a clinical endpoint. A surrogate biomarker is expected to predict clinical benefit (or lack of benefit, or harm) on the basis of epidemiological, therapeutic, pathophysiological, or other scientific evidence.

In the area of CNS, there are currently no agreed biomarkers (Kapur et al., 2012). This situation exists despite more than 50 years of neuropharmacological research into brain disease and in excess of 3 000 published research articles on biomarkers.

The FDA regularly updates guidance on biomarkers, but incentives for development of biomarkers seem to be few and limited unless they are tightly coupled to expensive treatments that are already on the market. This is often the case in the field of oncology, where treatments may cost up to $100 000 a year per patient. Given that biomarkers are generally perceived as a means to restrict the patient population for a drug or treatment, where drugs are relatively inexpensive, manufacturers may have concerns that a biomarker could have the effect of reducing the manufacturer’s revenue.

In addition to the lack of financial incentives to develop biomarkers, the fact that the field of brain research is perceived to be considerably more complex than that of oncology must also be considered. Basic CNS research and clinical understanding are not yet advanced enough to identify biomarkers or differentiate between the different subtypes of a condition. Furthermore, given that almost all psychiatric disorders are heterogeneous, biomarkers would need to cover a huge spectrum of disease. For this reason, biomarkers should relate to tractable and significant problems that impact on functional outcomes (e.g. impulsivity, compulsivity, episodic memory), rather than a disorder (e.g. ADHD, obsessive compulsive disorder, schizophrenia).

Having considered biomarkers, discussion turned to the application of alternative diagnostic tools. It was suggested that cognitive markers might feasibly be developed, although there were some concerns. Encouragingly, cognitive markers would be relatively cheap to develop. By linking such measures to the latest test developments in cognitive neuroscience, it will be possible to introduce highly valid and objective measures to replace older, subjective methodologies. Furthermore, while in certain situations cognitive markers would be surrogate endpoints, in others they constitute the core disability, as is increasingly recognized in the cases of schizophrenia, depression, and bipolar disorder, and has always been the case with respect to Alzheimer’s disease and other forms of age-related dementia. Again, there was general agreement that a large database would be essential to develop and test a cognitive marker.

Having proposed that a database could help drive forward the development of diagnostic and assessment tools, the elements that would make a successful database were then assessed. QoL is a required measurement for many modern clinical trials. Combining QoL data with other markers would ensure that treatments were not just efficacious according to assessment scales, but actually provided a real benefit to the patient.

There was general consensus that any prescribing for patients recruited to the database should be done in conjunction with a package of other measures, including psychotherapy, psychoeducation, the use of smart devices to monitor and improve adherence, and education of family members and caregivers. For example, combined pharmacological and psychological or cognitive treatments are likely to prove more effective than pharmacological treatments alone. Such a package would be more attractive to health authorities if a range of treatments were available and could be tailored to suit individual patients.

Although there was unanimous enthusiasm for a large-scale database, some barriers to the concept were identified. For example, lack of interest from the drug industry would mean that financial backing would have to be sought elsewhere. This could be overcome by offering incentives to the hospitals taking part in the scheme. Inducements would include financial savings associated with automated data collection, reduced paperwork, and rapid access to the database. Systems are already in existence that can link to a large database and provide outcome data for different interventions during a clinical consultation, thereby helping to optimize prescribing decisions.

Another barrier is the large number of records required to make a database worthwhile. The group estimated that between 50 000 and 100 000 patients would need to be recruited. It is worth noting that numbers approaching these have been achieved in large-scale studies of the genetic bases of schizophrenia and bipolar disorder (The International Schizophrenia Consortium, 2009). This has been accomplished through impressive levels of collaboration involving patient recruitment on a vast international scale. A large-scale database spanning several countries would face many challenges, including differing regulatory and legal requirements, but the group was confident that these could be met by building on the experience of existing databases.

Overall, it was felt that a large-scale database was central to improving patient outcomes, ensuring the relevance of outcomes measurement, and driving better science. Furthermore, there would be significant advocacy from clinicians, patients, caregivers, and patient groups. To augment the database and increase patient engagement, a patient website or social media registry could be set up. Again, regulatory constraints may be a barrier, but if the pharmaceutical companies themselves were not involved, the barriers would not be insurmountable, providing patient awareness/consent was fully considered and sought.

### Topic 5: Incentivizing Investment in Brain Research

Investment in serious brain disorders has lagged behind other fields of medicine in recent years. This is partly due to the negative attitudes towards brain disorders and in particular mental health disorders engendered by stigmatization. Initiatives such as The Year of the Brain, promoted by the European Brain Council (EBC), have gained significant support from organizations representing patients, healthcare professionals, and industry. However societal attitudes to people suffering from mental health disorders remain unsympathetic, with negative portrayals fueled by myths and misconceptions. As long as this situation remains, the prospect of increasing government funding and attracting investment in brain research is questionable. Fortunately, recent events may herald a significant change in attitude as increased support for brain research has been announced, e.g. investments of the National Institute of Health (NIH) across at least eight of its 27 institutes, and also focused initiatives, such as the Obama Brain Research through the Advancing Innovative Neurotechnologies Initiative, focusing on the brain connectome, or the €1 billion EU Brain initiative, the Human Brain Project. Another prominent initiative is the European Brain Prize, funded by Lundbeck with the intent of highlighting the importance of brain research and the prestige associated with this field.

Given the current economic climate—and the need for brain disorder research to compete for funds with other areas of medicine, such as cancer and HIV, which already have powerful and highly vocal lobby groups—it is imperative that action is taken now to remedy the situation and raise the profile of brain diseases as an area of huge unmet need. As already noted, the entire field of brain research and therapy continues to be hampered by the issue of stigma. The combination of stigma and the sheer complexity of brain research represents a major barrier to prioritization of brain diseases research by governments and investment by private companies.

Three key factors were in focus during the discussions:

- Increasing the perceived importance of brain research to governments, regulators, and society as a whole.
- Exploring ways of engaging with regulatory bodies to improve the visibility of potential returns to investors through clearer sub-categorized indications to differentiate products and via extending the period of exclusivity for CNS drugs.
- Driving investment in brain research to a level commensurate with its importance as an area of unmet medical need.

There was general agreement that raising awareness of brain diseases is a priority. However, there is also a need to provide payors with clear messages on the potential cost savings to society associated with improving treatment of brain diseases. To convey this message, cost-benefit analyses demonstrating the benefits of early detection and treatment are required. Governments are dependent on votes from the general public; hence, targeting public awareness of the key unmet needs in the area of brain disease will encourage governments, payors, and investors to fund drug development. In summary, it is crucial to be able to identify and engage with relevant government members and payors, promote potential savings from improved treatment of brain disease, and raise public awareness of the impact of mental illness.

On the issue of funding, it was agreed that brain diseases are not recognized by payors in the same way as other conditions, such as cardiovascular disease and

diabetes. In particular, the stigma associated with conditions such as schizophrenia and depression leads to a reluctance to seek medical help. This in turn results in brain disorders being under-recognized by healthcare professionals, payors, and governments. Furthermore, because people with serious brain disorders tend to be treated in a community setting over a long period of time, they are not perceived to represent a large direct cost to healthcare systems. The exact opposite is in fact the case, as they exert a long-term indirect burden on society in terms of healthcare associated with comorbidities, loss of productivity related to being unfit for work, the cost of care providers, and also loss of productivity and greater healthcare utilization by family members who have to give up work to provide care. These hidden, indirect follow-on costs are rarely considered when calculating the benefit of a particular drug or therapy and are a major weakness of HTA systems.

Funding of clinical research is also low; the underlying issue is that most public funding bodies do not prioritize research according to overall disease burden on society, preferring to give priority to other disease areas, such as oncology, where direct costs or disease burdens are easily recognized.

Against a background of limited resources for research, one key issue will be how to prioritize areas of research. The Roadmap for Mental Health Research in Europe (ROAMER), funded by the European Commission and scheduled for publication in September 2014, aims to develop a comprehensive and integrated mental health research agenda within the perspective of the EU Horizon 2020 program. ROAMER covers six major domains: infrastructures and capacity building, biomedicine, psychological research and treatments, social and economic issues, public health, and well-being.

There are major barriers to increasing funding and awareness of drug development for CNS diseases: governments and regulators do not understand the vast financial numbers involved, and the public do not understand the diseases and their impact on society. Brain diseases are often difficult to quantify, making it difficult to change perceptions and contributing to the reluctance of pharmaceutical companies to invest in drug development for brain disease. There is a need to address this from an economic perspective, including assessing the indirect costs of brain disorders, which are often particularly difficult to quantify. Governments are traditionally reluctant to commit to tackling an issue when they cannot quantify the short-term cost. Furthermore, budgets are often in silos in which health research is segregated from provision of healthcare services. Therefore, it would be necessary to engage with health, finance, and social affairs ministers to make them aware of the overarching need with regard to serious brain disorders.

It was noted that governments may be reluctant to be associated with issues, such as mental health, that are less well understood than cancers or cardiovascular diseases and which have a stigma attached. In the USA, certain government officials have linked brain disease with violent behavior, thus adding to existing stigma. Importantly, there is also a lack of patient advocacy; many patients with mental health issues are inherently incapable of speaking out for themselves and promoting their rights. Fundamental values should be adopted, including the right to receive compassion, respect, and love as well as medical care.

Furthermore, there is a perceptual divide between neurodegenerative and psychiatric disorders, which is somewhat ironic given that the first insights into neurodegeneration and dementia came from Alzheimer’s research in the Department of Psychiatry at the Ludwig-Maximilian University, Munich. Mood disorders and schizophrenia remain poorly understood, and in some quarters mental illness in general is naively regarded as self-inflicted. Degenerative disorders such as Parkinson’s disease and dementia are more accepted by governments and the general public, while at the same time the prominent cognitive and emotional symptoms that characterize these disorders remain in the background. Perhaps if the coexistence of cognitive and emotional disturbances, which are cardinal features of most neurodegenerative disorders, was recognized it would improve understanding of their true complexity and thereby help to de-stigmatize certain conditions.

When considering return on investment, it is important to engage the public as well as investors. It is essential to show investors and the general public the need for increased investment in brain disorder research, but also to demonstrate to investors the future value of cash flow with drug development. Potential benefits that would attract investment include higher than average peak sales of drugs, reduced time for new drugs to become available (e.g. rapid development time, targeted studies in specific populations, etc.), and/or lower cost or risk during drug development. Additionally, an extended exclusivity period would make a drug more attractive to investors. For instance, given longer than average development times, net present value calculations stand a greater chance of being positive, where a drug has an extended exclusivity and thereby sales period. Thus, investors could be encouraged to get involved.

Unfortunately, the current IP system and patent protection/market exclusivity clearly favors biologicals (in oncology and inflammation, for example). Due to the need to cross

the blood-brain barrier, CNS drugs tend to be small molecules, which are much simpler to imitate than biologicals, making rapid development and manufacture of similar competitor drugs relatively easy.

It is difficult for venture capitalists to assess return on investment due to the very long gap between investment and return. This prompts the question of whether a new investment scenario is required. Currently, governments (and subsequently pharmaceutical companies) are too focused on direct costs, to the detriment of indirect costs.

There was consensus among the group that there are too few opportunities for investment in the current drug pipeline (i.e. not enough new or interesting drugs to invest in) and an inadequate level of investment in research and new science to generate new ideas. This is particularly unfortunate given the explosion of new technologies in neuroscience and the innovative potential to use them to improve brain health. However, there was debate about whether this should be driven by industry or public funding. Oncology was cited as an area where publicly-funded research is significantly higher than for CNS diseases and, at the same time, pharmaceutical companies can identify opportunities to develop new drugs.

In contrast, there are very few incentives for venture capitalists to invest in brain diseases. Intelligent solutions are crucial to achieving increased funding. Simply comparing CNS with oncology in terms of their relative impact on the patient will not suffice. Such an emotive and negative message is more likely to reinforce existing attitudes and will do little to increase investment in brain research.

A campaign to inspire, equip, and inform is required to encourage investment. Given the lack of government engagement, there is a need for a joint investment strategy by companies who develop drugs and those who market drugs. An obvious target is public perception of mental health. It would be beneficial to raise awareness of the impact of mental disorders on workforce productivity and the subsequent cost to the economy. Concurrent efforts should be made to increase awareness and understanding of mental health issues and reduce stigma, both in the workplace and in society as a whole.

A new rationale for investment should be developed, whereby co-investment leads to greater productivity. An immediately identifiable barrier is the vast number of employer groups, necessitating a system for encouraging collective investment.

### Actions

1. Code of conduct dealing with conflict of interest issues,
2. Prevention, early diagnosis, and treatment,
3. Linking science and regulation,
4. Patient involvement in trial design, definition of endpoints, etc.,
5. Novel trial design,
6. Reproduction and confirmation of data,
7. Update of IP laws to facilitate repurposing and combination therapy (low priority),
8. Large-scale, global patient registries,
9. Editorials on nomenclature and biomarkers and diagnostic tools, and
10. Public awareness: brain disease advocates to attend G8 meetings and World Economic Forum Annual meetings in Davos, Switzerland.

### 1. Code of Conduct Dealing with Conflict of Interest Issues

#### Rationale

Public funding for research should be made conditional on the ability to demonstrate an understanding of regulatory requirements. Regulatory science training should be made available for students with an interest in continuing research and regulatory procedures. Cross-stakeholder groups, such as the CINP, should communicate with universities to promote the inclusion of regulatory science in the curriculum. Meanwhile, academic institutions such as the NIH should promote training and education that focuses on the understanding of regulatory science. Regulators should be encouraged to attend scientific conferences and engage with attendees on the subject of regulatory science. However, the involvement of regulators and payors is not guaranteed due to perceived or real conflict of interest issues. For example, the EMA had withdrawn from certain meetings, although it has subsequently relaxed its stance and has begun to collaborate with the ECNP. The absence of regulators from advisory committees and other key industry and academic meetings may have a detrimental effect on the dialogue between scientists and regulators and, in turn, hinder research efforts. In this context, conflict of interest represents a considerable barrier to the development of new treatments. To address the perceived issues of conflict of interest that may prevent regulators from attending meetings, such as the CINP Summit, representatives from industry and academia should engage with regulatory bodies to explore ways in which regulators can be included (learning from IMI and NIH consortia as well as from other activities where regulators and/or payors are represented on scientific/advisory board).

#### Goal

To develop a global code of conduct, making it possible for stakeholders in healthcare systems to work together and facilitate healthcare innovation.

#### Action

Initiation of a working party with representatives from patient and caregiver organizations, from industry, from scientific and medical organizations, and from payors—and jointly led by the PMDA, FDA, and EMA—to develop a code of conduct for any type of collaboration between the parties. The code should be globally accepted and should build on already-established codes in the healthcare area.

### Timeline:

Initiation of the working party during 2014 (reflecting the urgency of the unmet need) and adoption of a Healthcare Stakeholder Code of ethics by 2017.

### 2. Prevention, Early Diagnosis, and Treatment

#### Rationale

The spectrum of disorders of the brain is large, covering hundreds of disorders that are listed in either the mental or neurological disorder chapters of the established international diagnostic classification systems. These disorders have a high prevalence as well as short- and long-term impairments and disabilities. Therefore, they are an emotional, financial, and social burden to the patients, their families, and their social networks. Yet many of these disorders remain unrecognized, under-diagnosed, and generally poorly treated, leading to an increased burden on patients, caregivers, and society at large.

#### Goals

1. To reduce the number of people developing depression,
2. To provide support for people with schizophrenia living with their families and in their normal social context, and
3. To improve QoL of patients suffering from neurodegenerative disorders, ease the burden on caregivers, and delay nursing home admittance by diagnosing and treating early.

#### Actions

1. Depression: Undertake a major initiative to develop tangible recommendations to be implemented in human resources policies within the workplace. Recommendations should be developed in collaboration with relevant stakeholders, including employers and employee organizations, and should be modeled on the Europe-wide initiative “Target depression in the workplace” or Mental Health Canada (Wilkerson, 2014).
2. Schizophrenia: Undertake an initiative around early diagnosis and treatment to allocate social support to maintain patients with schizophrenia within the context of their family and social network, ideally allowing them to complete their education. The initiative could be based on the Danish Opus project. (Andreasen, Opus Project, see www.ispn-psych.org/docs/Opus_Project.pdf).
3. Neurological conditions: Initiate a public awareness campaign to make patients and caregivers/family members aware of the symptoms and the benefits of early diagnosis and treatment, including improved QoL and reduced burden on patients and caregivers alike.

#### Timelines

Initiatives to start in 2015 and be implemented as standard at a national level within a 5-year period.

### 3. Linking Science and Regulation

#### Rationale

Developing new prevention and treatment solutions requires a vibrant and integrated research ecosystem, comprising various sectors from industry and academia. To secure translation of research results into medical practice also requires early involvement of regulators and payors from the early stages of planning research, through execution of research programs, to uptake of results. Understanding of the opportunities offered by science and technology by the payors, regulators, and healthcare decision-makers on the one hand, and understanding of regulatory and legal requirements and constraints of healthcare decision-makers as well as clinicians on the other hand, is required to generate adequate data sets and, as a result, uptake of novel innovative therapeutic solutions. All the above should be underpinned by understanding patient perspective and needs. This virtuous cycle of investigators, decision-makers, patients, and an integrated research ecosystem across the value chain is essential for increasing research productivity and the probability of success in research and development (R&D).

#### Goal

To double the number of CNS drugs in the pharma development pipeline by 2018.

#### Action

Create an international forum on brain diseases involving the patient communities, FDA, EMA, PMDA, and representatives from the payor community and led by the CINP to facilitate:

1. Co-ordination of payor and regulatory requirements,
2. Accelerated translation of scientific breakthroughs into new endpoints and assessment tools acceptable to regulatory bodies, and
3. Assessment of the potential impact of scientific paradigm shifts and the latest scientific developments on clinical trial development.

#### Timeline

Work on establishing this forum should be initiated in 2014.

### 4. Patient Involvement in Trial Design, Definition of Endpoints, etc.

#### Rationale

Many concepts around patient-centered data are being explored. They include personalized medicine, patient-related outcomes, patient-centered healthcare, behavioral economics and health-plan design, real world benefit-risk analysis, coverage with evidence, and “big data.” The benefit-risk assessment in regulatory decision-making is under discussion in a number of settings, including: pre-approval data required for conditional approvals versus full marketing authorization; qualitative versus quantitative assessment methodology, and the need for active comparator clinical trials; post-approval data required to maintain the benefit-risk assessment; alignment on data required for regulatory benefit-risk assessment and health technology assessment needs; and communication of benefit-risk within medicine labels. The combination of patient-centric data and patient engagement could add value to R&D outputs and, as a result, to the healthcare systems. There should be a greater emphasis on patients’ functional outcomes and well-being. Benefit-risk assessments should be based on real-world improvements as reported by patients themselves. To this end, clinical trials should be designed that incorporate tools and methodologies to capture these patient-centric outcomes. New models should be sought for clinical studies that would satisfy the regulatory requirements of the FDA, EMA, and PMDA for approval of drugs. To improve the chances of success for innovative clinical trials, funding should be achieved via PPPs, and regulatory committees should be involved early in the development process. New trial methodologies should evaluate a whole treatment package, not just a drug. Treatment packages could include pharmacological, psychological, cognitive, and psychosocial interventions. Newer strategies, such as computer gaming to stimulate cognitive function, should also be included in the treatment package where appropriate. Not only patients themselves should give their perspectives on the benefit-risk assessment, but also care providers for children or patients with impaired insight or decision-making capabilities may choose to act as the decision-makers for the patients receiving the treatment. There is no clear evidence on when patients or a second party should make the decisions or provide input. But what is most lacking is sufficient real-time data. A key challenge is the deployment of these methodologies among patients enrolled in clinical trials.

#### Goals

1. To increase the relevance and impact of R&D, and
2. To improve translation and back-translation methodology.

#### Actions

1. Engage with leading national and international patient organizations within the scope of CINP activities to create a dedicated international platform,
2. Develop guidance for researchers on involvement of patients, regulators, payors, etc. in planning, execution, and exploitation of R&D, and
3. Engage with projects aiming at redefining benefit-risk assessment methodologies as well as collection of real-world data (e.g. IMI or Horizon 2020; see www.who.int/medicines/areas/priority_medicines/en/).

#### Timelines

Planning for these actions should start in 2014.

### 5. Novel Trial Design

#### Rationale

Clinical trials account for a large proportion of the overall development costs of any new medicine. Bayesian statistical methods are being used increasingly in clinical research to minimize the number of patients included in randomized, controlled trials and decrease the risk of patients receiving unfavorable treatment. The drive towards precision medicine is taking this concept even further, highlighting the need for the development of new patient-focused clinical outcome measures; new clinical trial paradigms to support the evaluation of benefit-risk in small numbers of stratified patient populations; and infrastructures for the collection and sharing of trial data, together with methods for meta-analysis of trial data to investigate outcomes across multiple trials in different locations. There is considerable interest in novel trial designs that could help reduce the high failure rate of late-stage clinical trials. An important aspect of these designs is the use of adaptive approaches, such as those based on Bayesian methodology. These designs can adapt an ongoing trial in response to information emerging from it while maintaining statistical rigor. Clinical trials should be designed that incorporate tools and methodologies to capture these patient-centric outcomes.

#### Goal

To decrease the number of patients and duration of clinical trials while increasing the robustness of evidence generated.

#### Actions

1. Mapping of adaptive design initiatives to identify gaps that can be addressed through collaborative research in the space of brain diseases and new models that would satisfy regulatory and payor requirements,
2. Engage regulators and clinicians in dialogue to explore ways to speed up acceptance of adaptive trials,
3. Engage with electronic health records/IT community to explore ways to strengthen and harmonize the data collection and processing infrastructure (including quality and standards of data), and
4. Promote adaptive trial design in the clinicians’ community.

#### Timelines

Planning of this activity should start in 2014.

### 6. Reproduction and Confirmation of Data

#### Rationale

Reproducibility is the foundation of all modern research, the standard by which scientific claims are evaluated. Drug manufacturers rely heavily on early-stage academic research and can waste millions of dollars on products if the original results are later shown to be unreliable. Patients may enroll in clinical trials based on conflicting data, and sometimes see no benefits or suffer harmful side effects. A recent study suggests that success rate of Phase II trials fell to 18% in recent years (Arrowsmith, 2011), and some link the lack of reproducibility to the decline in Phase II success rates. Some PPPs where academic and public partners validate each other’s results in real time may offer a solution in some cases, but these are exceptions and cannot be applied as routine practice in all cases.

#### Goal

To increase the overall quality of scientific and clinical data.

#### Action

Engage with publishers and public and private funders of research to discuss potential solutions to the lack of reproducibility.

#### Timelines

The dialogue with stakeholders should be initiated in 2014.

### 7. Update of IP Laws to Facilitate Repurposing and Combination Therapy

#### Rationale

There are currently only limited economic incentives for innovators to fully explore the potential of new drugs, as timelines and costs for conducting clinical trials, combined with limited exclusivity periods, make it non-viable from a business perspective. Nor are there clear economic or regulatory incentives to invest in repurposing of old products that lost their exclusivity periods (and are “genericized”) or combinations of products (old-old, new-new, old-new).

#### Goal

To facilitate research on novel and old compounds to fully exploit their therapeutic potential.

#### Action

To map current incentives versus unmet needs to identify opportunities/gaps within the current system. This work should be carried out by a PPP combining the efforts of academics, industry, patients, regulators, and payors. As a global organization, the CINP is well positioned to lead this activity.

#### Timelines

Kick off an analysis (co-funded with other parties, possibly industry associations) in 2014/2015.

### 8. Large Scale, Global Patient Registries

#### Rationale

Registries that enroll patients with a specific disease or who have received a particular treatment are an important source of data for patient-centered outcomes research. In addition to providing clinically relevant data that are meaningful to patients and providers, registries are known for their ability to provide data on populations not typically studied in clinical trials (e.g. children, elderly, minorities, pregnant women, and those with multiple co-morbidities). Registries can offer adaptable designs and data collection strategies, making them particularly useful when treatments are rapidly changing. Because of their non-experimental design (i.e. no randomization), registries can be used to examine the impact of physician practice behaviors on quality of care, prescribing preference, and other important but difficult to quantify co-variates. Good design and use of registries, however, requires strong understanding of both the potential for bias that threatens all observational studies and the methodological and operational tools that can be used to minimize the influence of such biases. Therefore, patient registries are essential both for optimizing healthcare delivery and for speeding up research processes (in particular for patient stratification, clinical trials recruitment). These either do not exist, or the quality and accessibility of information is poor. The infrastructures are not in place in all countries.

#### Goal

To set up high-quality patient registries in priority areas.

#### Action

Define an action plan based on mapping and analysis of current registries for filling the gaps. The action plan should be run as a collaborative project between patient organizations, industry, and the public health systems, potentially as a PPP.

#### Timelines

Discussions should be initiated between stakeholders on how to structure this endeavor in 2015.

### 9. Editorials on Nomenclature, Biomarkers, and Diagnostic Tools

#### Rationale

The biological basis for most brain diseases is poorly developed and understood, and thus starting points for drug R&D are generally lacking. Furthermore, a less than helpful update of the DSM makes it important to specify general diagnostic criteria, ideally with a link to biological features and criteria for developing biomarkers for brain diseases. This would facilitate the opportunity to develop rationally-designed drugs for brain disorders.

#### Goal

To have globally accepted nomenclature for brain diseases, ideally linked to defined biologies and criteria for biomarkers for brain diseases.

#### Actions

1. An editorial on common nomenclature was published in 2014. Professor Zohar (ECNP, Israel) was responsible for this action as an important part of the effort to accelerate CNS drug discovery (Zohar et al., 2014).
2. An editorial for publication in 2014/2015 outlining the usefulness of biomarkers in psychiatry and the development of other types of diagnostic tools, including cognitive and electrophysiological markers. This action will be led by Professor Kapur (King’s College London) and Dr Zoran Simic (Medicines and Healthcare Products Regulatory Agency).

#### Timeline

To be completed in 2014–2015.

### 10. Public awareness—Brain Disease Advocates to Attend G8 Meetings and World Economic Forum Annual Meetings in Davos, Switzerland

#### Rationale

The true burden of brain disorders is generally not recognized in our societies. Brain disorders, especially psychiatric disorders, have historically been stigmatized, and in some cultures not even recognized as disease; at the same time, much of the burden of brain disorders falls outside traditional healthcare systems, typically impacting wider social systems. Data from the WHO (see www.who.int/medicines/areas/priority_medicines/en/) and the EBC (Wittchen et al., 2011) indicate that the economic burden on society of these disorders surpasses any other disease area. In fact, data from the EBC document shows that the accumulated costs of brain disorders are higher than those for cancer and cardiovascular disease combined (Nutt and Attridge, 2014). Given that a healthy workforce is vital to the innovation, drive, and growth of a society, the importance of raising awareness around brain disorders cannot be underestimated.

#### Goals

1. To promote the recognition and prioritization of brain disorders, and
2. To stimulate investment in brain disorders across healthcare systems and in basic research, commensurate with the societal burden of these conditions.

#### Actions

1. Initiation of global awareness campaigns led by the CINP, EBC, and WHO, and
2. Attendance of brain disorder advocates at G8 and WEF meetings beginning in 2017.

#### Timelines

Discussions to be initiated in 2014.

## Conclusion

CNS diseases, and in particular, mental health disorders, are a growing health challenge of the 21^st^ century. Currently, at least 10% of the global population is affected by a mental health disorder (Patel and Saxena, 2014), with that figure set to increase year on year. Meanwhile, the rate of development of new CNS drugs has not increased for many years, despite unprecedented levels of investment.

In response to the above situation, the CINP convened a Summit to discuss ways of developing innovative partnerships to accelerate CNS drug discovery. The objectives of the Summit were to explore the issues affecting the value chain in brain research and gain insights from all key stakeholders leading to identification of achievable objectives to address these issues. Following these discussions, action plans have been developed to bring about measurable improvements across the value chain and accelerated CNS drug discovery.

### Next Steps

As a follow up to the 2013 CINP Summit Meeting, a Summit Update was held in June 2014 in Vancouver, Canada. The purpose of this meeting was to prioritize the actions from the CINP Summit and agree on the implementation of a series of initiatives. The attendees of this meeting included the authors of this paper, plus other key figures from within the CNS research arena, such as regulators, research funders, pharmaceutical industry representatives, and members of patient advocate organizations. This consortium of thought leaders was tasked with implementing the following actions:

#### Code of Conduct

- Initiation of a working party with representatives from all stakeholder groups—and jointly led by the PMDA, FDA, and EMA—to develop a code of conduct for any type of collaboration between the parties. The code should be globally accepted and should build on already established codes in the healthcare area. Initiation of the working party to take place during 2014 (reflecting the urgency of the unmet need) and development of a Healthcare Stakeholder Code of Ethics by 2017.

#### Public Awareness

- Developing a public/private awareness campaign with a global outreach, led by the CINP, EBC, and WHO. The campaign will be directed towards 100 key public figures in business and government over a three-year timeline. It will be accompanied by initiatives aimed at the general public.
- Developing tangible recommendations to target depression in the workplace. Recommendations will be developed in collaboration with relevant stakeholders, including employers and employee organizations, and will be modeled on the Europe-wide initiative “Target depression in the workplace” or the Mental Health Canada program (Wilkerson, 2014). This initiative should be started in 2014/2015 and be implemented as standard at a national level within five years.
- Identifying advocates to attend the G8 and WEF meetings to raise the profile of brain disorders, create awareness of their huge burden on society, and discuss the need for investment in brain disorders across healthcare systems and in basic research, commensurate with the societal burden of these conditions. Attendance at G8 and WEF should be secured by 2017.

#### Public–Private Partnership

- Developing a PPP to fund the design of a new clinical trial model incorporating tools and methodologies to capture patient-centric outcomes, such as QoL and daily functioning, while still meeting the regulatory requirements of the FDA, EMA, and PMDA for the approval of drugs. Planning for this action should begin in 2015.

## Statement of Interest

Dr Phillips served until July 2013 on the board of directors of Allon Therapeutics; declares a patent pending related to the use of D-Govadine (PCT/CA2012/050526); and declares a pending patent (PCT/CA2004/001813) for an IV formulation of the interference peptide Tat-GluA23Y. Dr Sahakian consults for Cambridge Cognition, Servier and Lundbeck; holds a grant from Janssen/J&J; and has shares in CeNeS and share options in Cambridge Cognition. Dr Krishnan has multiple patents pending and licensed intellectual property owned by his employer; has direct and indirect interests in Orexigen, Corcept, and Atentiv; is Chairman of SCRI an academic CRO held by Ministry of Health Holdings Singapore; and is Chairman of NMRC and Board Member of HSA. The views expressed do not necessarily reflect the opinions of these organizations.

Dr Race is a research analyst (financial analyst) at Deutsche Bank AG and does not have any financial relationships with any of the organizations mentioned in the report, with any of the other authors’ organizations, or with any other organization that might have an interest in the submitted work. The views expressed in this document do not necessarily reflect the opinions of Deutsche Bank.
